# Effects of using primary percutaneous coronary interventions on the incidence of new‐onset atrial fibrillation following an acute myocardial infarction

**DOI:** 10.1002/clc.24167

**Published:** 2023-10-25

**Authors:** Li‐Hua Pan, Bo‐Yu Yan, Jing Zhu, Qi Lu, Jie Hui

**Affiliations:** ^1^ Department of Cardiology Affiliated Hosptial of Nantong University Nantong China; ^2^ Department of Cardiology Pingxiang People's Hosptial Pingxiang China; ^3^ Department of Cardiology The First Affiliated Hospital of Soochow University Suzhou China

**Keywords:** acute myocardial infarction, atrial fibrillation, coronary interventions, new‐onset atrial fibrillation

## Abstract

**Background:**

Acute ST‐segment elevation myocardial infarction (STEMI) and new‐onset atrial fibrillation (AF) are associated with increased risk of mortality.

**Hypothesis:**

This study aimed to determine the proportion of patients who go on to develop new‐onset a AF after undergoing a primary or delayed percutaneous coronary intervention (PCI) for an acute STEMI and to explore possible risk factors.

**Methods:**

One hundred and fifty‐four patients who underwent PCI after STEMI were included in the study. Patient characteristics, baseline blood tests and cardiac parameters, type of PCI, and incidence of new‐onset AF within 3 months of PCI were recorded and analyzed.

**Results:**

Fifteen developed new‐onset AF following the PCI, and 139 patients maintained a sinus rhythm. Univariate analysis showed significant differences between the two patient groups in terms of age, nature of the PCI (primary vs. delayed), left atrial diameter, and left ventricular diastolic dysfunction (*p* < .05). Age (odds ratio [OR] = 1.065, 95% confidence interval [CI]: 1.007–1.127, *p* < .05) and left atrial diameter (OR = 1.165, 95% CI: 1.008–1.347, *p* < .05), were independent predictors of new‐onset AF after PCI. Primary PCI (OR = 0.232, 95% CI: 0.066–0.814, *p* < .05) was an independent protective factor.

**Conclusion:**

Age and left atrial diameter were independent risk factors of new‐onset AF in patients undergoing a PCI following an acute myocardial infarction, while primary PCI was a protective factor. This discovery can help reduce mortality rate, improve long‐term prognosis, and provide a theoretical basis for the prevention of new‐onset AF.

## Graphical abstract

Using primary percutaneous coronary interventions (PCIs) on the incidence of new‐onset atrial fibrillation (AF) following an acute myocardial infarction (AMI). Age and left atrial diameter were independent risk factors of new‐onset AF in patients undergoing a PCl following an AMI, while primary PCI was a protective factor.

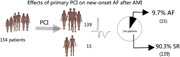

## 1 INTRODUCTION

Acute ST‐segment elevation myocardial infarction (STEMI) is a critical disease in cardiovascular medicine that affects human health.^1^ An acute myocardial infarction (AMI) can damage the sinus and atrioventricular nodes resulting in atrial fibrillation (AF), which can arise as a result of problems with electrical conductivity.^2^ New‐onset AF during hospitalization is an independent risk factor for in‐hospital mortality in AMI,^3^ and it is also correlated with an increase in overall 30‐day mortality following an AMI.^4^ However, the mechanism underlying these associations remains unclear. Current literature suggests that the pathogenesis of new‐onset AF in patients with an AMI is driven by factors such as acute left atrial dilatation, which is caused by a rise in atrial pressure.^5^ New‐onset AF is considered a secondary complication of AMI, and the prognosis is poor when it occurs independently.^6^, ^7^

Primary percutaneous coronary interventions (PCIs) for AMIs can reduce infarct size, improve cardiac function, and reduce short‐term and long‐term mortality.^8^ However, there is little research on whether it can significantly improve the prognosis of acute STEMIs that are complicated by new‐onset AF.

There are limited data available on the incidence and prognosis of new‐onset AF in AMI patients who had undergone a PCI in the Chinese population. Therefore, it is important to quantify the burden of this complication and find effective methods to reduce it. The aim of this study was to ascertain the incidence rate of new‐onset AF in patients who had undergone a PCI following a STEMI and to identify any risk factors that predicted this complication.

## 2 MATERIALS AND METHODS

Two hundred patients who had undergone a PCI following a STEMI in the coronary heart disease care unit (CCU) in the Affiliated Hospital of Nantong University between August 2017 and July 2019 were identified retrospectively. Data relating to the patients' baseline characteristics and their clinical variables were extracted from their medical records. Patients with a history of AF and those who had died in the CCU were excluded from the study. In addition, patients younger than 18 years or older than 85 years were excluded, as were patients with severe valvular disease, congenital heart disease, and those with a pre‐existing coronary artery bypass. Patients with a history of cardiac surgery, thyroid disease, pulmonary heart disease, and those who were taking antiarrhythmic drugs at the time of admission were also excluded. In total, 46 patients were excluded from the study, and 154 patients were enrolled.

All patients had a baseline record of their cardiac rhythm before undergoing the PCI. The widely accepted definition of AMI, was used to confirm the diagnosis of AMI and to further classify it as a STEMI or a non‐ST‐segment elevation myocardial infarction.

Patients undergoing a primary PCI (The patients who were suitable for primary PCI were performed via radial artery or femoral artery. They were considering acute coronary syndrome includes the following types: 1. Patients with persistent chest pain, ECG indicates ST‐segment elevation; 2. Patients with severe heart failure or cardiogenic shock suitable for vascular reconstruction; 3. Patients with moderate to large myocardial infarction at risk and evidence of failure of thrombolytic therapy; 4. 3–24 h after thrombolytic therapy was started in patients with stable hemodynamics and evidence of successful thrombolytic therapy) were routinely given 300 mg oral aspirin, 180 mg ticagrelor, and 40 mg atorvastatin before the procedure, and 2500 U of heparin was administered through the sheath before coronary angiography. During the operation, we used heparin according to the patient's weight, generally 100 U/kg, followed by 1000 U heparin was added every hour during the operation, and guidelines for basic medication were given after the operation. Patients undergoing a delayed PCI (defined as the PCI not being delivered over 24 h after symptom onset, the PCI being delivered after symptoms of chest pain had subsided, or the patient receiving the PCI 7–10 days following presentation due to being unable to undergo a primary PCI) were treated with conventional medication. These included anticoagulants and antiplatelet drugs, statins, angiotensin‐converting enzyme inhibitors, and beta‐blockers based on the patient's blood pressure readings and heart rate. The cardiac rhythm of the patient was monitored using a 12‐lead electrocardiogram (ECG) or a Holter monitor for 2–5 days following the PCI, and the findings were reviewed by experienced cardiologists. If the patient had arrhythmia, the ECG was reviewed immediately. During the follow‐up period of 3 months, patients were regularly monitored using ECGs or a Holter monitor if they presented with symptoms, and new‐onset AF was diagnosed where appropriate.

AF was considered to be present on a tracing when no P waves could be identified, a visible F wave was present accompanied by vibration or severe tremor in the baseline, and the R–R interval was irregular. A diagnosis of new‐onset AF was confirmed if the following criteria were met: AF had not been detected on admission and there was no history of persistent atrial flutter or paroxysmal AF, at least 30 s of AF were identified by an experienced cardiologist either from Holter monitoring data or on an ECG,^9^ and the AF was first detected after the PCI.

The management of AF was carried out in accordance with current international guidelines (2017 h/EHRA/ECAS/APHRS/SOLAECE expert consensus), taking into account the patient's existing medication regimen and the outcome of the PCI.^10^ This study was approved by the Ethics Committee of the Affiliated Hospital of Nantong University, and informed consent was obtained from all enrolled patients.

The following patient data were collected on admission: age, sex, and whether the patient had a pre‐existing diagnosis of hypertension or diabetes mellitus. Additionally, the following data on cardiac function were collected on admission: the location of the infarct‐related artery (IRA), the left ventricular ejection fraction, the presence of left ventricular diastolic dysfunction (LVDD), and the left atrial diameter. The following serum levels were also checked before the PCI: total cholesterol(TC), triglycerides, high‐density lipoprotein cholesterol, low‐density lipoprotein cholesterol, serum creatinine, and N‐terminal pro‐B‐type natriuretic peptide.

The IBM SPSS Statistics software (version 25.0, IBM Corporation) was used for the analysis, and the data were expressed as a percentage (%). The differences between the groups were measured using the chi‐square test. The measurement data were expressed as mean ± standard deviation, and the difference between the groups was determined using the independent sample *t* test and a nonparametric test. Logistic regression analysis was used to determine the independent risk factors for new‐onset AF in patients following an AMI. The two‐tailed test was used, and *p* < .05 was considered statistically significant.

## 3 RESULTS

Out of the 154 patients included in the study, 106 (68.9%) were males, and the mean age was 65.22 ± 11.92 years. Hypertension and diabetes mellitus were found in 89 patients (57.8%) and 27 patients (17.5%), respectively. A STEMI had been diagnosed in all patients, and these were further subdivided into left anterior descending artery infarctions (74 patients, 48.1%), right coronary infarctions (62 patients, 40.3%), and left circumflex artery infarctions (18 patients, 11.7%). Primary PCIs were conducted in 113 patients (73.4%), and 41 patients (26.6%) underwent a delayed PCI. Of the 154 patients in the study, 15 (9.7%) developed new‐onset AF, while 139 (90.3%) displayed sinus rhythm. The demographic and clinical characteristics of patients are listed in Table 1, which shows the baseline characteristics of the patients with new‐onset AF and those with sinus rhythm. No significant differences were seen between patients with new‐onset AF and those with sinus rhythm in terms of sex, hypertension, diabetes mellitus, and IRA. However, significant differences were observed between them in relation to age and whether the PCI was primary or delayed (*p* = .008; *p* = .014; Table 1).

**Table 1:** Baseline characteristics of 154 study patients.

Variable	Total sample	New‐onset AF (*n* = 15)	Sinus rhythm (*n* = 139)	*p*
Age (years)	65.22 ± 11.92	72.93 ± 10.17	64.39 ± 11.83	.008
Male gender (%)	106 (68.9)	9 (60.0)	97 (69.8)	.437
Hypertension (%)	89 (57.8)	8 (53)	81 (58)	.713
Diabetes mellitus (%)	27 (17.5)	1 (6.7)	26 (18.7)	.419
Infarct‐related artery (%)				.231
LAD	74 (48.1)	10 (67)	64 (46)	
LCX	18 (11.7)	2 (13)	16 (12)	
RCA	62 (40.3)	3 (20)	59 (42)	
Primary PCI (%)	113 (73.4)	7 (46.7)	106 (76.3)	.014

Abbreviations: AF, atrial fibrillation; LAD, left anterior descending artery; LCX, left circumflex artery; RCA, right coronary; PCI, percutaneous coronary intervention.

Table 2 shows the baseline characteristics of the patients with primary PCI and those with delayed PCI. No significant differences were seen between patients with primary PCI and those with delayed PCI in terms of age, sex, hypertension, diabetes mellitus, and IRA. However, significant differences were observed between patients who developed new‐onset AF and those who did not (*p* = .014; Table 2).

**Table 2:** Baseline characteristics of primary PCI group and delayed PCI group.

Variable	Total sample	Primary PCI (*n* = 113)	Delayed PCI (*n* = 41)	*p*
Age (years)	65.22 ± 11.92	64.90 ± 1.082	66.10 ± 2.051	.584
Male gender (%)	106 (68.9)	76 (67.3)	30 (73.2)	.484
Hypertension (%)	89 (57.8)	61 (54.0)	28 (68.3)	.112
Diabetes mellitus (%)	27 (17.5)	22 (19.5)	5 (12.2)	.294
Infarct‐related artery (%)				.054
LAD	74 (48.1)	49 (43.4)	25 (61.0)	
LCX	18 (11.7)	12 (10.6)	6 (14.6)	
RCA	62 (40.3)	52 (46.0)	10 (24.4)	
New‐onset AF (%)	15 (73.4)	7 (6.2)	8 (19.5)	.014

Abbreviations: AF, atrial fibrillation; LAD, left anterior descending artery; LCX, left circumflex artery; RCA, right coronary; PCI, percutaneous coronary intervention.

**Figure 1:**
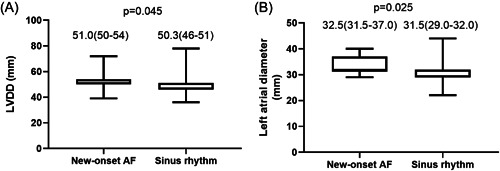
(A) The relationship between left ventricular diastolic dysfunction (LVDD) and new‐onset atrial fibrillation (AF). Of the 154 patients included, 15 patients developed new‐onset AF, and 139 patients maintained a sinus rhythm. A statistically significant difference in LVDD was observed between patients with new‐onset AF and those with sinus rhythm (*p* = .045). (B) The relationship between left atrial diameter and new‐onset AF. Of the 154 patients included, 15 patients developed new‐onset AF, and 139 patients maintained a sinus rhythm. A statistically significant difference in the left atrial diameter was observed between patients with new‐onset AF and those with sinus rhythm (*p* = .025).

According to the analysis performed, there was a statistically significant difference in left atrial diameter (*p* = .025) and LVDD (*p* = .045) between patients who developed new‐onset AF and those who did not (Figure 1). The baseline characteristics of the patients with new‐onset AF are presented in Table 3.

**Table 3:** Test results and echocardiography of 154 study patients.

Variable	New‐onset AF (*n* = 15)	Sinus rhythm (*n* = 139)	*p*
TC (mmol/L)	4.27 (3.69–4.79)	4.71 (4.03–5.31)	.173
TG (mmol/L)	1.06 (0.75‐2.48)	1.65 (1.11–2.51)	.116
HDL‐C (mmol/L)	1.01 (0.89–1.14)	1.12 (0.93–1.26)	.227
LDL‐C (mmol/L)	2.82 ± 0.64	3.08 ± 0.66	.239
SCr (mmol/L)	87.9 (64.4–93.0)	74.7 (63.4–91.1)	.354
NT‐pro BNP (ng/L)	3276 (1325–4352)	1540 (374–2622)	.055
Left atrial diameter (mm)	32.5 (31.5–37.0)	31.5 (29.0–32.0)	.025
LVEF (%)	54 (41–60)	55 (50–60)	.395
LVDD (mm)	51.0 (50–54)	50.3 (46–51)	.045

Abbreviations: LVDD, left ventricular end‐diastolic diameter; LVEF, left ventricular ejection fraction; SCr, serum creatinine; TC, total cholesterol; TG, triglyceride.

Multivariate logistic regression analysis was performed to determine whether any factors were independent predictors of whether a patient would go on to develop new‐onset AF. The results showed that LVDD alone (odds ratio [OR] = 1.060; 95% confidence interval [CI]: 0.981–1.146, *p* > .05) was not an independent predictor for the development of new‐onset AF after the PCI. Age (OR = 1.065; 95% CI: 1.007–1.127, *p* < .05) and left atrial diameter (OR = 1.165; 95% CI: 1.008–1.347, *p* < .05) were independent predictors for patients developing new‐onset AF following the PCI. The PCI being primary was also an independent predictor of risk, significantly reducing the incidence of new‐onset AF compared to delayed PCIs (OR = 0.232; 95% CI: 0.066–0.814, *p* < .05) (Table 4).

**Table 4:** Binary logistic regression analysis of predictors of new‐onset atrial fibrillation.

Variable	OR	95% CI	*p*
Age	1.065	1.007–1.127	.028
Left atrial diameter	1.165	1.00–1.347	.039
Primary PCI	0.232	0.066–0.814	.023
LVDD	1.060	0.981–1.146	.138

*Note*: Value was used as continuity.

Abbreviations: CI, confidence interval; LVDD, left ventricular diastolic dysfunction; OR, odds ratio; PCI, percutaneous coronary intervention.

## 4 DISCUSSION AND CONCLUSION

This retrospective study showed that increasing age and a wider left atrial diameter were independent risk factors for AMI patients developing new‐onset AF following PCI, while the PCI being primary rather than delayed was a protective factor.^11^

During the acute period of myocardial infarction, the rapid and irregular ventricular rate associated with AF can aggravate coronary blood flow problems, thereby worsening heart function and resulting in a poor prognosis. Studies have shown that the incidence of new‐onset AF after myocardial infarction is between 7% and 21%.^12^ Rathore found that the mortality of AMI patients who developed new‐onset AF after admission was significantly higher during hospitalization and follow‐up compared to both patients with persistent AF and patients who maintained a sinus rhythm following myocardial infarction.^5^ Jabre also found that the overall mortality rate of patients with AMI complicated by new‐onset AF increased by 40% compared with the mortality of AMI patients with sinus rhythm.^13^ The prognosis of AMI with AF is worse than the prognosis of AMI without AF, with a high risk of future stroke, and the prognosis of new‐onset AF is worse than that of AMI.^14^ Therefore, reducing the incidence of new‐onset AF in patients with AMI can reduce patient mortality and improve long‐term prognosis.

A STEMI usually develops rapidly and has a high mortality rate. The timely resolution of the coronary artery occlusion is key to treating a STEMI successfully. Early, full, and continuous opening of the IRA is vital to myocardial recovery. It improves the blood circulation in the myocardial ischemic site, protecting the heart function of the patient and improving the prognosis.^15^ Primary PCIs can effectively reduce myocardial ischemic injury, reduce infarct size, improve cardiac function, and reduce short‐ and long‐term mortality. It has been reported that it can significantly improve the conversion rate of new‐onset AF in STEMI patients to sinus rhythm.^6^ A delayed PCI refers to a patient undergoing reperfusion therapy 7–10 days after the initial onset of symptoms, and during this delay, patients receive routine drug treatment. Studies have shown that delayed PCIs can improve left ventricular remodeling and myocardial systolic function in STEMI patients, and although it cannot salvage the ischemic myocardium within the optimal time, it can still benefit patients.^16^ No reports are available on whether primary PCIs are an important factor in the prognosis of AMI.

In this study, 41 patients underwent delayed PCI, primarily due to delayed treatment times and conservative opinions from patients and their families. Most of them missed the best time to perform primary PCI due to lack of knowledge about the disease and fear of emergency surgery. The results of this study showed that the incidence of new‐onset AF in the primary PCI group was significantly lower than in the delayed PCI group (*p* < .05). Multivariate logistic regression analysis showed that age and left atrial diameter were independent risk factors for new‐onset AF in patients with a STEMI, while a primary PCI was an independent protective factor. The latter finding may be related to early revascularization; the early opening of occluded coronary arteries can rapidly improve left atrial ischemia and hypoxia, improve the arterial blood supply of the sinus node, and reduce the occurrence of chronic congestive heart failure,^17^ thereby reducing the incidence of AF. Age and left atrial diameter may be related to an inflammatory etiology, which could contribute to the increased risk of new‐onset AF following a PCI for a STEMI.^18^ Further studies are required to determine the specific mechanisms that link these factors to new‐onset AF.

This retrospective study had some limitations. The specific onset time and duration of new‐onset AF could not be determined; hence, it was not classified into episodic, paroxysmal, persistent, or permanent AF. In addition, the sample size of the study was small, and further research on larger samples from more centers is warranted in the future to clarify the relevant etiology. What is more, we hope that in future research, the basic information of patients can be provided as objective as possible, such as wearable devices or electronic watches that can detect heart rhythms.

In summary, age and left atrial diameter were shown to be independent risk factors for new‐onset AF in patients who had undergone a PCI following a STEMI, while primary PCIs were shown to significantly reduce the incidence of new‐onset AF and improve the prognosis of STEMIs. In this study, the factors associated with patients developing new‐onset AF after undergoing a PCI for a STEMI were explored to determine possible methods for reducing the mortality rate, improving the long‐term prognosis, and with a view to providing a theoretical basis for the prevention of new‐onset AF.

## CONFLICT OF INTEREST STATEMENT

The authors declare no conflict of interest.

## Data Availability

The data sets used and analyzed during the current study are available from the corresponding author on reasonable request.
